# Direct cell reprogramming for tissue engineering and regenerative medicine

**DOI:** 10.1186/s13036-019-0144-9

**Published:** 2019-02-13

**Authors:** Alexander Grath, Guohao Dai

**Affiliations:** Department of Bioengineering, Northeastern University, Lake Hall 214A, 360 Huntington Avenue, Boston, MA 02115 USA

**Keywords:** Cell reprogramming, Transdifferentiation, Gene editing, Epigenetics, Stem cells, Tissue engineering

## Abstract

Direct cell reprogramming, also called transdifferentiation, allows for the reprogramming of one somatic cell type directly into another, without the need to transition through an induced pluripotent state. Thus, it is an attractive approach to develop novel tissue engineering applications to treat diseases and injuries where there is a shortage of proliferating cells for tissue repair. In certain tissue damage, terminally differentiated somatic cells lose their ability to proliferate, as a result, damaged tissues cannot heal by themselves. Examples of these scenarios include myocardial infarctions, neurodegenerative diseases, and cartilage injuries. Transdifferentiation is capable of reprogramming cells that are abundant in the body into desired cell phenotypes that are able to restore tissue function in damaged areas. Therefore, direct cell reprogramming is a promising direction in the cell and tissue engineering and regenerative medicine fields.

In recent years, several methods for transdifferentiation have been developed, ranging from the overexpression of transcription factors via viral vectors, to small molecules, to clustered regularly interspaced short palindromic repeats (CRISPR) and its associated protein (Cas9) for both genetic and epigenetic reprogramming. Overexpressing transcription factors by use of a lentivirus is currently the most prevalent technique, however it lacks high reprogramming efficiencies and can pose problems when transitioning to human subjects and clinical trials. CRISPR/Cas9, fused with proteins that modulate transcription, has been shown to improve efficiencies greatly. Transdifferentiation has successfully generated many cell phenotypes, including endothelial cells, skeletal myocytes, neuronal cells, and more. These cells have been shown to emulate mature adult cells such that they are able to mimic major functions, and some are capable of promoting regeneration of damaged tissue in vivo. While transdifferentiated cells have not yet seen clinical use, they have had promise in mice models, showing success in treating liver disease and several brain-related diseases, while also being utilized as a cell source for tissue engineered vascular grafts to treat damaged blood vessels. Recently, localized transdifferentiated cells have been generated in situ, allowing for treatments without invasive surgeries and more complete transdifferentiation. In this review, we summarized the recent development in various cell reprogramming techniques, their applications in converting various somatic cells, their uses in tissue regeneration, and the challenges of transitioning to a clinical setting, accompanied with potential solutions.

## Introduction

Cellular reprogramming has become possible in recent years due to several advances in genetic engineering, where cellular DNA can be manipulated and reengineered with mechanisms such as transgenes, transcription activator-like effector nucleases (TALENs), zinc finger nucleases (ZFNs), and CRISPR/Cas9 [1]. In typical cellular reprogramming, cells are first converted into an induced pluripotent stem cell (iPSC) state and are then differentiated down a desired lineage to generate a large quantity of reprogrammed cells [2]. The introduction of several key transcription factors converts somatic cells into stem-like cells that propagate indefinitely and differentiate into most cell types in the body. Thus, these cells show great potential for uses in clinical applications, such as tissue engineering, disease modeling, and drug discovery. The major downside of iPSC reprogramming is the lengthy time commitment involved in the reprogramming and differentiation processes, as it usually takes several months and involves significant cost. Another problem is the potential for cancerous tumor formation when the reprogrammed iPSCs do not fully differentiate into their final cell types. As such, clinical iPSC treatments are met with adversity from government bodies that regulate medical procedures and drugs. Another method of reprogramming has emerged whereby somatic cells of one type can be directly converted into another somatic cell type without the need for the iPSC step; this is referred to as direct cell reprogramming or transdifferentiation. The process of transdifferentiation does not require cell division, and thus reduces the risk of mutations and tumor formation, making it more viable for clinical applications when compared to iPSC reprogramming. Additionally, because the pluripotent state is avoided, the transdifferentiation process is generally shorter than iPSC reprogramming, making them more appealing for uses in time-sensitive clinical settings [3]. This review will discuss the various methods used to transdifferentiate cells, targeted cell phenotypes, the current uses and applications of transdifferentiated cells in regenerative medicine and tissue engineering, and challenges associated with clinical translations and proposed solutions.

## Direct cell reprogramming techniques and mechanisms

Cellular reprogramming can be achieved through multiple methods, each with their own advantages and disadvantages. The reprogramming process generally includes introducing or upregulating key reprogramming factors that are vital for the development of cellular identity and function. Cells used in the transdifferentiation process are mature somatic cells. These cells do not experience an induced pluripotent state, and therefore the chance of tumorigenesis is drastically reduced. Transdifferentiation can occur in three major ways. First, exogenous transgenes can be introduced into cells to overexpress key transcription factors to kickstart the transdifferentiation process [4–7]. Secondly, endogenous genes vital to the transdifferentiation process can be specifically targeted and silenced or upregulated, using methods that focus on the direct manipulation of DNA or the epigenetic environment, such as CRISPR/Cas9 [8–11]. Lastly, transcription pathways can be targeted with pharmacological agents that can induce an immunological response in cells [12], causing a cascade that triggers epigenetic remodeling, or directly alter the epigenetic environment [13, 14]. The use of viral vectors to introduce exogenous transgenes into cells is currently the most prominent method to induce transdifferentiation, but this method has been shown to be relatively inefficient. On the other hand, upregulating endogenous genes results in much higher conversion efficiencies, which opens the door for using the transdifferentiated cells in large-scale applications [8].

### Exogenous transgene overexpression

Viruses have been the staple method of introducing foreign genetic material into a host cell for decades and have undergone thorough research. As such, it is not surprising that they have emerged as one of the most common ways to introduce transgenes into cells in order to drive transdifferentiation. In fact, the original work that generated iPSCs was done using viral vectors [2]. Broadly, lentiviruses and retroviruses see the most use in transdifferentiation studies due to their ability to effectively integrate DNA directly into the genome of the host cell [15]. The host cell will begin to produce proteins from the viral DNA, and the viral DNA will be passed on to the daughter cells during cell division. One notable difference between lentiviruses and retroviruses is that lentiviruses are capable of infecting both non-dividing and dividing cell types while retroviruses are only able to infect the latter [16]. Lentiviral vectors have a small carrying capacity and are unable to carry large segments of DNA, inhibiting their use to overexpress genes that are long in length [5].

Non-integrating viruses have also been examined for their ability to drive the transdifferentiation process. Generally, these methods are met with efficiencies that are much lower than those achieved when utilizing lentiviruses or retroviruses, as the transdifferentiation process either takes longer to produce the same yield or generates fewer viable reprogrammed cells. Both adenoviruses and Sendai viruses have been used in transdifferentiation studies [6, 7]. Adenoviruses insert the transgene such that it is transiently expressed and Sendai viruses replicate in the cytosol. Meng et al. (2011) generated functional neurons from fibroblasts using adenoviral vectors with an efficiency of 2.7%, while Vierbuchen et al. (2010) used lentiviral vectors to achieve an efficiency of 7.7% [5, 17]. Sendai viruses have not seen widespread usage, likely due to their incredibly low efficiency rates [18].

**Fig. 1:**
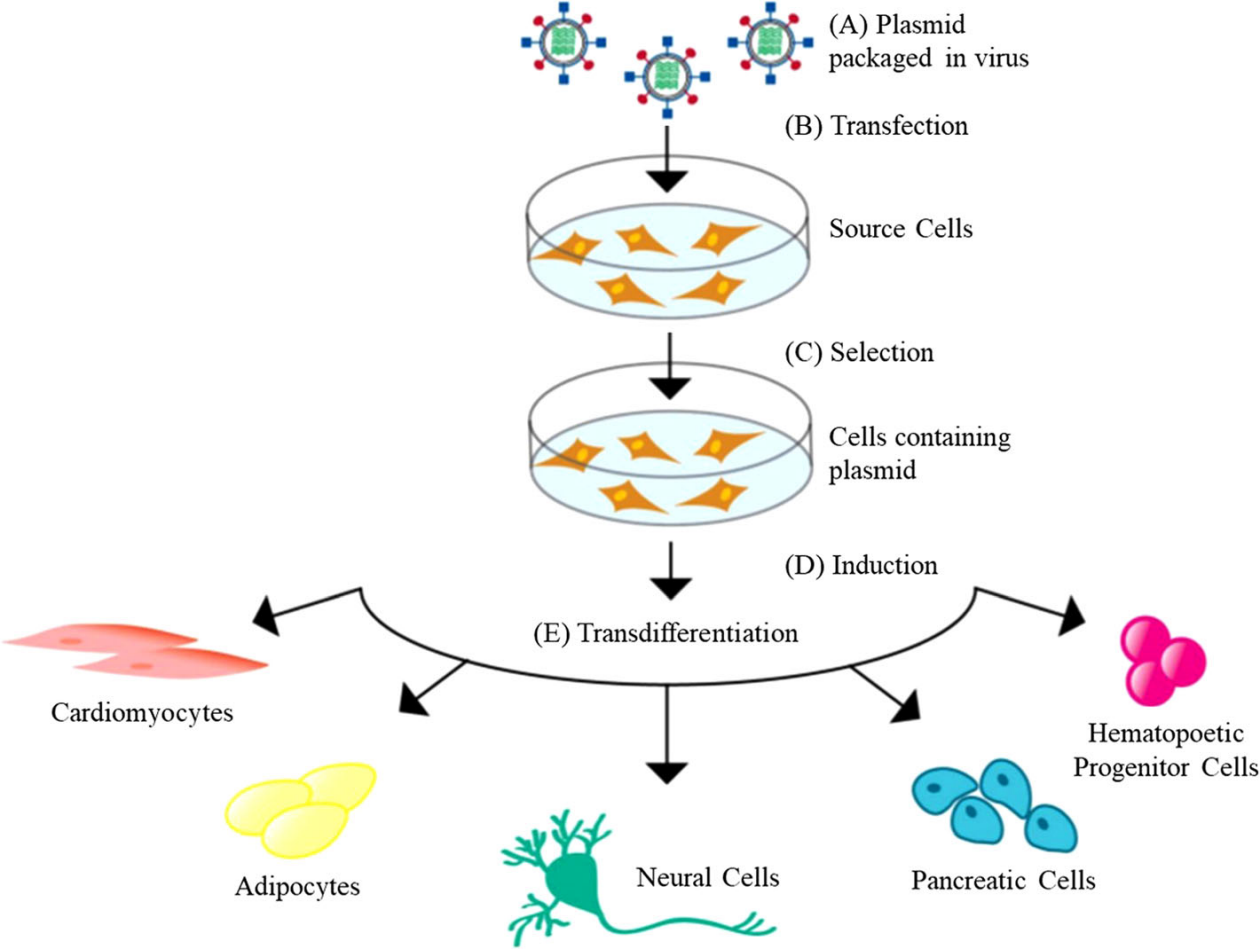
Basic transdifferentiation protocol via viral transgene overexpression [19]

Regardless of the type of virus being used, they are designed to overexpress exogenous transcription factors (TFs). TFs are responsible for regulating gene expression by controlling the rate of transcription, allowing for the upregulation or downregulation of certain genes. Additionally, they are in charge of directing cell division, growth, differentiation, and migration throughout a cell’s lifecycle. By regulating these TFs, it is possible to give cells new characteristics. In effect, the TFs manipulate widespread gene expression, allowing for the cells to change function and resemble another cell type. Hence, to begin the virus-directed transdifferentiation process, the coding DNA for select TFs are first cloned into plasmids and packaged into a virus (Fig. 1a). The plasmids typically have a region coding for antibiotic resistance to allow for selection after transfection. Cells are infected with the virus (Fig. 1b), selected using antibiotics (Fig. 1c), and begin to transcribe the TF coding DNA as if it were its own (Fig. 1d). The TFs are drastically overexpressed, causing changes in the expression of downstream genes and thus driving the cell to pursue a desired lineage (Fig. 1e) [19]. To control the expression of the TF, the TF coding DNA is typically cloned into a plasmid that is transcribed in the presence or absence of tetracycline, referred to as Tet-On and Tet-Off plasmids, respectively [20].

The main challenge when inducing transdifferentiation is choosing what TFs to overexpress. Many studies use a guess-and-check method, where TFs are chosen based on logical conclusions. For example, TFs that are active during the development of a cell type or drive the differentiation of stem cells into a specific cell type are often investigated first [4, 8, 21–24]. The TFs’ potential to transdifferentiate cells into a desired type is evaluated both individually and in conjunction with other TFs, as the overexpression of several TFs together could potentially drive the transdifferentiation process to be quicker and more efficient than individual TFs.

Due to the low efficiency often achieved by strictly targeting TFs that play a role in the development of a certain cell type, Margariti et al. (2012) first overexpressed Oct4, Sox2, KLF4, and c-Myc (OSKM) in cells, in an effort to “prime” and prepare the cells for the transdifferentiation process [25]. By introducing OSKM to the cells before adding differentiation media, the cells enter a partial-iPSC (PiPSC) state and are transdifferentiated directly into endothelial cells while completely removing the risk for tumor formation in vivo. Cells derived from this PiPSC method had a reprogramming efficiency of roughly 34%, which is much higher than similar studies that did not create PiPSCs before generating endothelial cells through viral-directed transdifferentiation (6.8% [4], ~ 16% [26]).

Transgenes can be introduced into cells through other non-integrating, non-viral methods such as transient transfection and electroporation [27]. These methods express the transgene temporarily, are met with efficiency problems, and are not commonly used in recent transdifferentiation studies. These techniques follow a protocol similar to the viral transdifferentiation protocol.

### Endogenous gene regulation

#### Silencing endogenous genes with CRISPR/Cas9

Direct genomic editing is feasible with the discovery of CRISPR/Cas9. CRISPR/Cas9 was originally a bacterial defense system, but this system has been adapted to allow for the insertion of a short DNA sequence at any desired destination in the human genome. This is done through the use of guide RNA (gRNA), which is necessary for CRISPR/Cas9 binding. In short, gRNA is a strand of 20 nucleotides that allows the CRISPR complex to specifically bind to DNA that matches the sequence of the gRNA. Its ability to recognize and bind to incredibly specific sequences of DNA with limited off-target effects makes it a promising method for the future of transdifferentiation [9].

CRISPR/Cas9 can be used to induce transdifferentiation by permanently silencing specific genes in cells. gRNA is designed to target a certain gene that needs to be silenced, and the CRISPR complex will find the gene and make a double stranded DNA break, thereby disrupting the gene. It can interfere with the DNA repair process and prevent the gene from repairing itself properly. Thus, the gene is effectively knocked out and the cell will no longer express it. Wang et al. (2017) used CRISPR/Cas9 to permanently knockout the Myod1 gene in mouse myoblasts to drive transdifferentiation towards adipose cells [28]. In a slightly different vein, CRISPR/Cas9 can also be used to augment the normal transdifferentiation process. For example, Rubio et al. (2016) employed CRISPR/Cas9 to directly convert fibroblasts into neuropathological-resistant neuronal cells. CRISPR/Cas9 was used to silence the TSC2 gene in fibroblasts, which, when mutated, plays a major role in the onset of tuberous sclerosis. The fibroblasts were then transduced with lentiviral vectors to overexpress Ascl1, Lmx1a, and Nurr1, which promote the transdifferentiation process that converts fibroblasts into neuronal cells [10]. Overall, CRISPR/Cas9 can be used to drive or aid the transdifferentiation process, either by silencing genes to drive transdifferentiation or being used in conjunction with other techniques to create disease-resistant cells.

#### Upregulating endogenous genes with dCas9

While CRISPR/Cas9 is used to silence a gene by breaking double stranded DNA, a mutant form of Cas9 can be utilized to perform different functions. One such mutant is dCas9, a nuclease-deactivated version of CRISPR/Cas9 that binds to, but does not break, DNA. Therefore, it can be used to enhance or suppress the expression of endogenous genes in order to promote the transdifferentiation process. dCas9 can upregulate silenced genes with the help of fused transactivator proteins to unwrap complex chromatin structures and recruit transcription complexes to promote the expression of the normally silenced gene. Chakraborty et al. (2014) used dCas9 fused with the transactivator protein VP64 to upregulate the Myod1 gene in fibroblasts to create skeletal myocytes. Myod1 is well known to kickstart the transdifferentiation process that drives fibroblasts into skeletal myocytes and causes a cascade of other skeletal myocyte-specific markers to be upregulated [8]. This study shows the promise of using dCas9 to replace current mainstream exogenous overexpression methods, and there is much ongoing research focusing on transdifferentiation using dCas9.

**Fig. 2:**
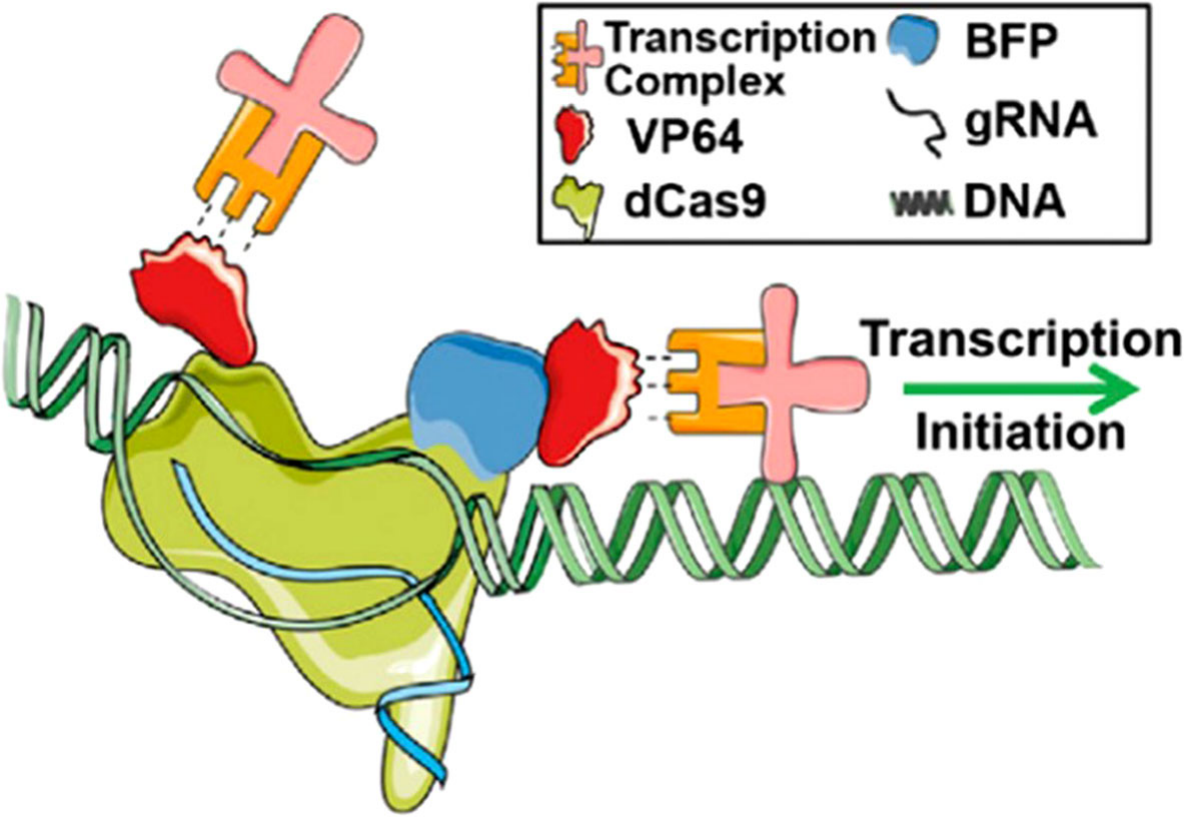
Schematic of dCas9-VP64. dCas9 binds to the promoter region of the target gene, then uses VP64 to recruit transcription factors to initiate the transcription of the gene [8]

Additionally, dCas9 complexes can be coupled with fusion proteins to improve performance. Transactivator proteins and repressor domains can be utilized to enhance or suppress gene expression, respectively, with transactivator proteins seeing more use in reprogramming. Common fusion proteins include VP64, VP64-p65-Rta (VPR), histone acetyltransferases (HATs), synergistic activation mediators (SAMs), and SunTag [8, 11, 29–31]. VP64 is a transactivator domain that hires transcription factors to help the dCas9 complex upregulate the gene of interest. The basic structure of a fluorescently-labelled dCas9-VP64 complex can be seen in Fig. 2. VPR domains contain VP64 but also include two other transcription factors. In effect, all three of these transcription factors are targeted to the same gene, vastly improving its upregulation in comparison to VP64 alone [11]. HATs, such as p300 and CREB-binding proteins, are enzymes capable of acetylating lysine residues found on histones. Once they become acetylated, the DNA wrapped around the histones is loosened, allowing dCas9 to better access the DNA. dCas9 uses the HAT domain to expose the DNA, then binds to the promoter region of the gene of interest and recruits transcription factors to upregulate the gene [29]. The use of SAMs is much more direct; instead of altering histone acetylation, SAMs simply contain three components (MS2, p65, and HSF1) that help recruit a wide array of transcription factors. SAMs have the ability to upregulate genes greatly, as the recruited transcription factors work synergistically in order to activate the gene of interest [30]. SunTag is an activator system that utilizes a repeating polypeptide array that recruits several copies of the same antibody. The polypeptide array is attached to a VP64 domain that is fused to dCas9. Transcription factors are bound to the antibodies that target the polypeptide array, effectively carrying transcription factors directly to the CRISPR complex, and allowing for the efficient upregulation of a select gene [31].

### Pharmacological agents

Sayed et al. (2014) discovered that the lentiviral vectors used to transdifferentiate cells do more than just deliver transgenes to target cells; the viruses themselves also cause widespread changes in gene expression and epigenetic modifiers, through the activation of innate immune signaling pathways, notably Toll-like receptor 3 (TLR3). Viral double stranded DNA is responsible for the stimulation of TLR3, which then downregulates innate histone deacetylases and upregulates HATs. These epigenetic changes specifically targeted endogenous genes that are a vital part of the pluripotency network. Sayed et al. introduced polyinosinic:polycytidylic acid (Poly I:C) to stimulate TLR3 in human foreskin fibroblasts in an effort to generate endothelial-like cells. Roughly 2% of the cells treated with Poly I:C expressed CD31, a key endothelial protein responsible for adhesion and monolayer formation. Once isolated, these cells were capable of mimicking select endothelial cell functions, including the ability to produce nitric oxide, express endothelial-specific markers, and form a typical “cobblestone” morphology that is a hallmark of endothelial cells [12].

Cells have been treated with pharmacological agents that are capable of modifying the genetic and epigenetic environment in order to promote transdifferentiation. Kaur et al. (2014) reprogrammed fibroblasts into skeletal myocytes using 5-azacytidine, a DNA methyltransferase inhibitor [13]. 5-azacytidine is a chemical analog of cytidine. Cells metabolize azacytidine in a cascade of reactions, ultimately incorporating it into DNA by binding it to guanine. However, due to differences in molecular structure, azacytidine is unable to be methylated, thus inhibiting DNA methylation [32]. The inhibition of DNA methylation leads to a change in the epigenetic environment, resulting in a change in gene expression. Cardiac cells treated with 5-azacytidine showed skeletal myocyte properties, including the upregulation of Myod1, a skeletal myocyte-specific marker, changes in morphology, and the emergence of multinucleated myotubes [13]. Another DNA methylation inhibitor, zebularine, functions similarly to 5-azacytidine, except it controls the differentiation of murine mesenchymal stem cells into cardiomyocytes [14]. DNA methylation inhibitors pose serious threats, however; they become cytotoxic in large concentrations, making it difficult to effectively reprogram cells while maintaining viability. Another type of pharmacological agent used for transdifferentiation is dexamethasone, a glucocorticoid that is capable of activating certain transcription factors to promote the transdifferentiation of several cell types [33–35]. Dexamethasone binds to glucocorticoid receptors, which promotes changes in gene expression [36].

## Current uses of Transdifferentiation techniques

### Cell sources

Transdifferentiation requires source cells to be converted into reprogrammed cells. The source cells used in transdifferentiation studies vary, but, generally, the cells are readily available and found in abundance in an adult human body. As such, fibroblasts are a prominent choice. Fibroblasts are the most common type of cells found in connective tissue and are responsible for producing extracellular matrix and collagen. Due to their abundance, they can be easily obtained from patients via a minimally invasive skin biopsy, making them ideal candidates to transdifferentiate into patient-specific cells [21]. Various types of fibroblasts have been used, including neonatal, adult dermal, and adult lung. Neonatal fibroblasts are thought to be easier to reprogram because they are at an earlier stage of the developmental hierarchy [37]. Dermal fibroblasts are one of the easiest types of fibroblasts to acquire, but they are also one of the most difficult to reprogram due to their reluctance to change into other cell types [38]. There are disadvantages associated with fibroblasts; they tend to be heterogeneous and need to be expanded, which could give rise to random mutations [27]. Amniotic cells are also a popular choice for transdifferentiation, for a similar reason to neonatal fibroblasts [26]. A list of cell sources, along with generated cell types and corresponding reprogramming factors are summarized in Table 1.

**Table 1:** Summary of reprogramming factors and transdifferentiated cell types

Cell Source	Transdifferentiation Method	Target Cell Type	Reprogramming Factors	References
Human Adult Dermal Fibroblast	Viral Vectors	Neurons	Brn2, Mty1l, miRNA-124	Ambasudhan et al. (2011) [22]
Human Adult Peripheral Blood Mononuclear Cells	Electroporation		Ascl1, Brn2, Myt1l, Ngn2	Tanabe et al. (2018) [27]
Human Striatum Astrocytes	Viral Vectors		Ascl1, Brn2, Myt1l	Torper et al. (2013) [40]
Murine Embryonic and Postnatal Fibroblasts	Viral Vectors		Ascl1, Brn2, Myt1l	Vierbuchen et al. (2010) [17]
Murine Bone Marrow Stromal Cells	Pharmacological Agents		Dimethylsulphoxide, butylated hydroxy-anisole, KCl, valproic acid, forskolin, hydrocortisone, insulin	Zurita et al. (2008) [41]
Human Neonatal Fibroblasts	Viral Vectors	Hepatocytes	Foxa2, Hnf4α, C/EBPβ, c-Myc	Kogiso et al. (2013) [23]
Human Embryonic Fibroblasts	Viral Vectors		Hnf1α, Hnf4α, Foxa3	Huang et al. (2014) [24]
Murine Pancreatic Cells	Pharmacological Agents		Dexamethasone, oncostatin M	Shen et al. (2003) [33]
Human Adult Fibroblasts	Viral Vectors	Endothelial Cells	ETV2	Morita et al. (2014) [4]
Murine Amniotic Cells	Viral Vectors		Sox17	Schachterle et al. (2017) [26]
Human Newborn Dermal and Lung Fibroblasts	Viral Vectors Pharmacological Agents		Oct4, Sox2, KLF4, c-Myc bFGF, βME	Margariti et al. (2012) [25]
Human Newborn Foreskin Fibroblasts	Pharmacological Agents		Polyinosinic:polycytidylic acid	Sayed et al. (2015) [12]
Murine Embryonic Fibroblasts	Pharmacological Agents	Skeletal Myocytes	5-azacytidine	Kaur et al. (2014) [13]
Murine Embryonic Fibroblasts	CRISPR/dCas9		Myod1	Chakraborty et al. (2014) [8]
Human Dermal Fibroblasts	Viral Vectors Pharmacological Agents		Myod1 SB431542, Chir99021, EGF, IGF1	Boularaoui et al. (2018) [58]
Human Dermal Fibroblasts	Pharmacological Agents	Chondrocytes	Cartilage-derived morphogenetic protein 1	Yin et al. (2010) [61]
Mouse Dermal Fibroblast	Viral Vectors		c-Myc, KLF4, Sox9	Outani et al. (2013) [62]
Murine Adult Pancreatic Exocrine Cells	Viral Vectors (in situ)	Pancreatic β-Cells	Pdx1, Ngn3, Mafa	Zhou et al. (2008) [64]
Human Pancreatic Exocrine Cells	Viral Vectors		MAPK, STAT3	Lemper et al. (2015) [65]
Murine Cardiac Fibroblasts	Viral Vectors (in situ*)*	Cardiomyocytes	Gata4, Mef2c, Tbx5	Qian et al. (2012) [75]
Murine Bone Marrow Mesenchymal Stem Cells	Pharmacological Agents		5-azacytidine, Zebularine	Naeem et al. (2013) [14]
Murine Cardiac Fibroblasts	Pharmacological Agents		miRNA-1, miRNA-133, miRNA-208, miRNA-499	Jayawardena et al. (2015) [85]
Murine Myoblasts	CRISPR/Cas9	Adipocytes	Myod1	Wang et al. (2017) [28]
Human Skeletal Muscle Fibroblasts	Pharmacological Agents		Dexamethasone, 1-methyl-3-isobutylxanthine, PPARγ agonists	Agley et al. (2013) [34]
Human Subcutaneous Adipocytes	Pharmacological Agents	Osteoblasts	Calcitriol, dexamethasone, ascorbic acid, and beta-glycerophosphate	Justesen et al. (2004) [35]
Murine Adipose Tissue-Derived Stem Cells	Viral Vectors		Runx2	Zhang et al. (2006) [86]
Murine Preadipocytes	Viral Vectors		Runx2, MKP-1	Takahashi et al. (2011) [87]

### Target cell phenotypes

#### Neuronal cells

**Fig. 3:**
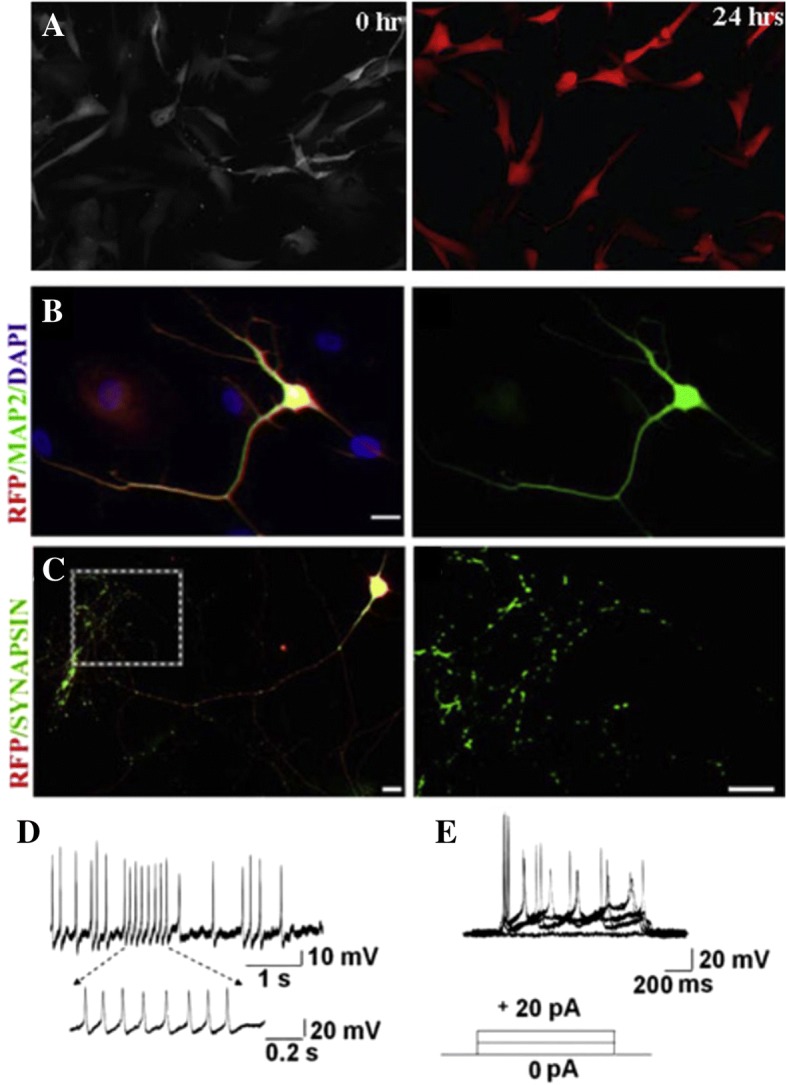
**a** Cell morphology at Day 0 (left) and Day 18 (right) after induction. **b** Immunofluorescent staining of MAP2 (green). **c** Immunofluorescent staining of synapsin-1(green). **d** Traces of spontaneous action potentials in the reprogrammed cells. E) Repetitive trains of evoke action potentials in the reprogrammed cells [22]

Unsurprisingly, neuronal cells are one of the most popular targets for transdifferentiation, due to their limited supply and limited regeneration potential in vivo. Ambasudhan et al. (2011) discovered that overexpressing Brn2 and Mytl1 in conjunction with microRNA-124 in fibroblasts generated neuronal-like cells [22]. Brn2 is important for neuronal commitment as well as the generation of autonomic neurons. Mytl1 helps in developing the nervous system in stem cells that are being differentiated into neuronal cells [39]. These induced neurons experienced a change in morphology over the course of 18 days (Fig. 3a). They expressed MAP2 (Fig. 3b), a marker of mature neuronal cells, as well as synapsin-1 (Fig. 3c), indicating the presence of mature, functional synapses between the induced neuronal cells. Roughly 15% of the cells showed spontaneous action potentials (Fig. 3d), and approximately 20% sustained repeated bursts of evoked action potentials (Fig. 3e). Additionally, more sources of neuronal cells were discovered by overexpressing these factors in other cell types, such as astrocytes, pericytes, and hepatocytes [40]. Similar results have also been achieved using bone marrow stromal cells and mesenchymal stem cells [41, 42].

As an alternative to fibroblasts, Tanabe et al. (2018) have used human adult peripheral blood mononuclear cells as well as T-lymphocytes to generate induced neuronal cells, showing that terminally differentiated, mature human cells can be transdifferentiated into a distant lineage efficiently [27]. The blood cells were transfected with Brn2, Ascl1, Myt1l, and Ngn2 vectors to drive transdifferentiation [43–45]. Over 3 weeks, the blood cells drastically changed morphology to resemble neuronal cells. The conversion process was later enhanced by culturing the cells with select media supplements, notably a bone morphogenic protein pathway blocker (dorsomorphin), a TGF-β pathway inhibitor (SB431542), and an adenylyl cyclase activator (forskolin). All three of these compounds increased the yield of neuronal cells substantially [27, 46].

#### Hepatocytes

**Fig. 4:**
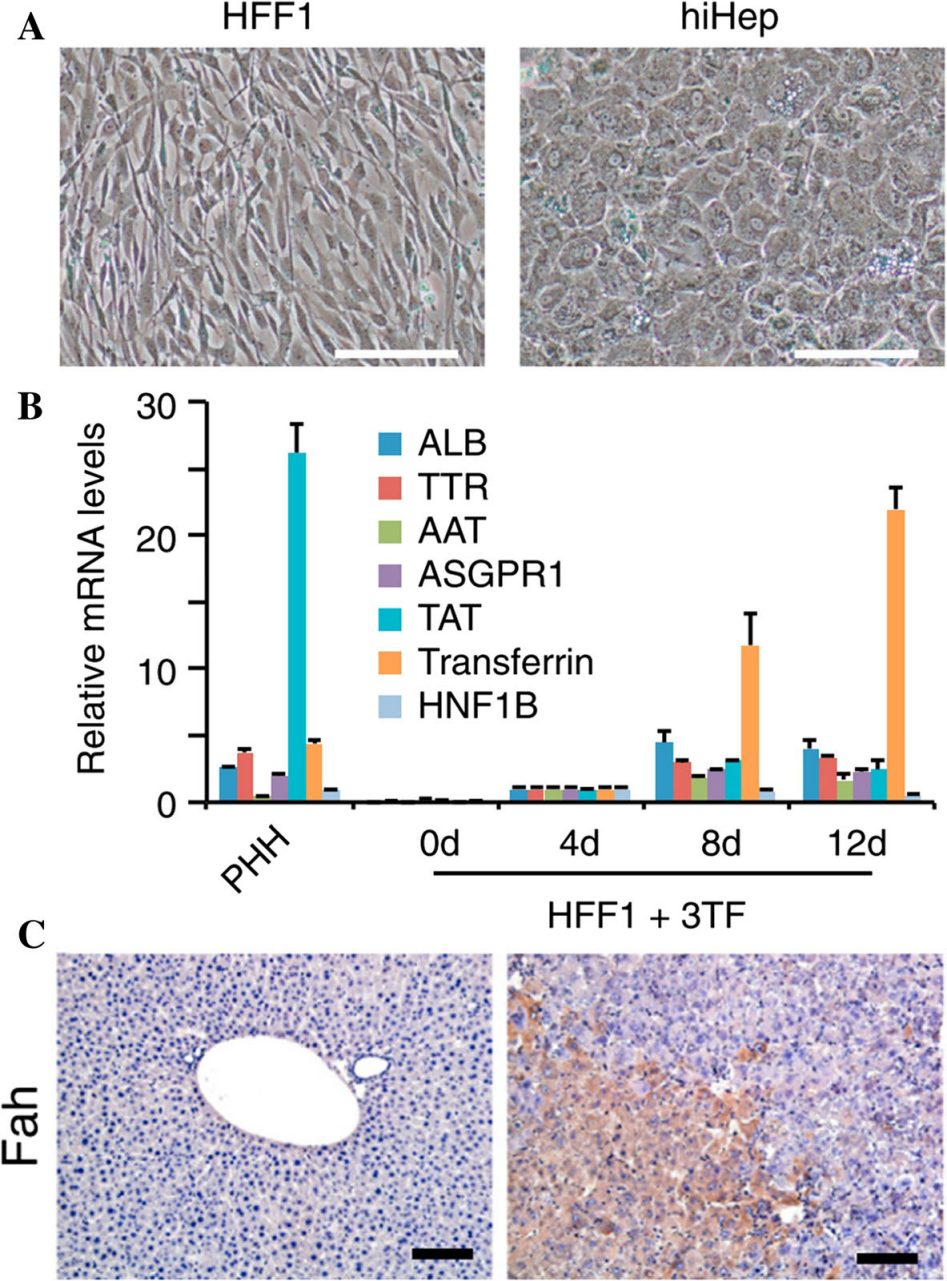
**a** Cell morphology of fibroblasts (HFF1) and hiHeps. **b** Hepatocyte marker qRT-PCR analysis of HFF1 transduced with Hnf1α, Hnf4α, and Foxa3 (3TF), compared to hepatocytes (PHH). **c** Staining of Fah in F/R mice, without (left) and 9 weeks after implantation with hiHep (right) [24]

Hepatocytes are also an attractive cell type to create using transdifferentiation. By introducing transgenes to overexpress Hnf1α, Hnf4α, and Foxa3 in human embryonic fibroblasts, Huang et al. (2014) created cells that closely resembled hepatocytes [24, 47, 48]. These human induced hepatocyte (hiHep) cells were subjected to rigorous functional testing, in which they resembled normal hepatocytes in terms of morphology (Fig. 4a) and mRNA marker profiles (Fig. 4b). The hiHep cells were then transplanted into knockout mice, where they regenerated the liver and restored liver function in roughly 50% of the mice (Fig. 4c) [48]. The same lab group continued this work and developed hepatic stem cells from fibroblasts. These cells were able to differentiate into both cholangioblasts and hepatocytes, which are responsible for liver bile secretion and low-density lipoprotein production, and are both extremely prominent in the liver, making up roughly 70–85% of its mass. The induced hepatic stem cells were implanted into mice and successfully restored and regenerated damaged liver [24].

Kogiso et al. (2013) achieved similar results by overexpressing c-Myc, Foxa2, Hnf4α, and C/EBPβ in human neonatal and forehead fibroblasts. The induction of these factors drove morphological changes within 3 days. The gene expression profile of the induced hepatocytes revealed that they produced albumin, a function vital to liver cells [23]. The general consensus is that the overexpression of Hnf4α and Hnf1α in conjunction with Foxa1, 2, or 3 is sufficient to drive the transdifferentiation of fibroblasts into hepatocyte-like cells [24]. This process was further developed and refined by also targeting the transcription factor Kdm2b, which promoted greater conversion efficiencies as well as more prominent hepatocyte features [49].

In place of fibroblasts, pancreatic cells have also been explored as a source for generating functional hepatocytes. Shen et al. (2003) successfully transdifferentiated murine pancreatic cells into hepatocyte-like cells using dexamethasone and oncostatin M, which both play a role in activating C/EBPs [33, 50]. These cells undergo drastic morphological changes and express hepatocyte-specific proteins. Additionally, the transdifferentiated cells performed key hepatocyte functions, such as storing glycogen and secreting albumin [33, 51]. This study has not yet been replicated in human cells.

#### Endothelial cells

**Fig. 5:**
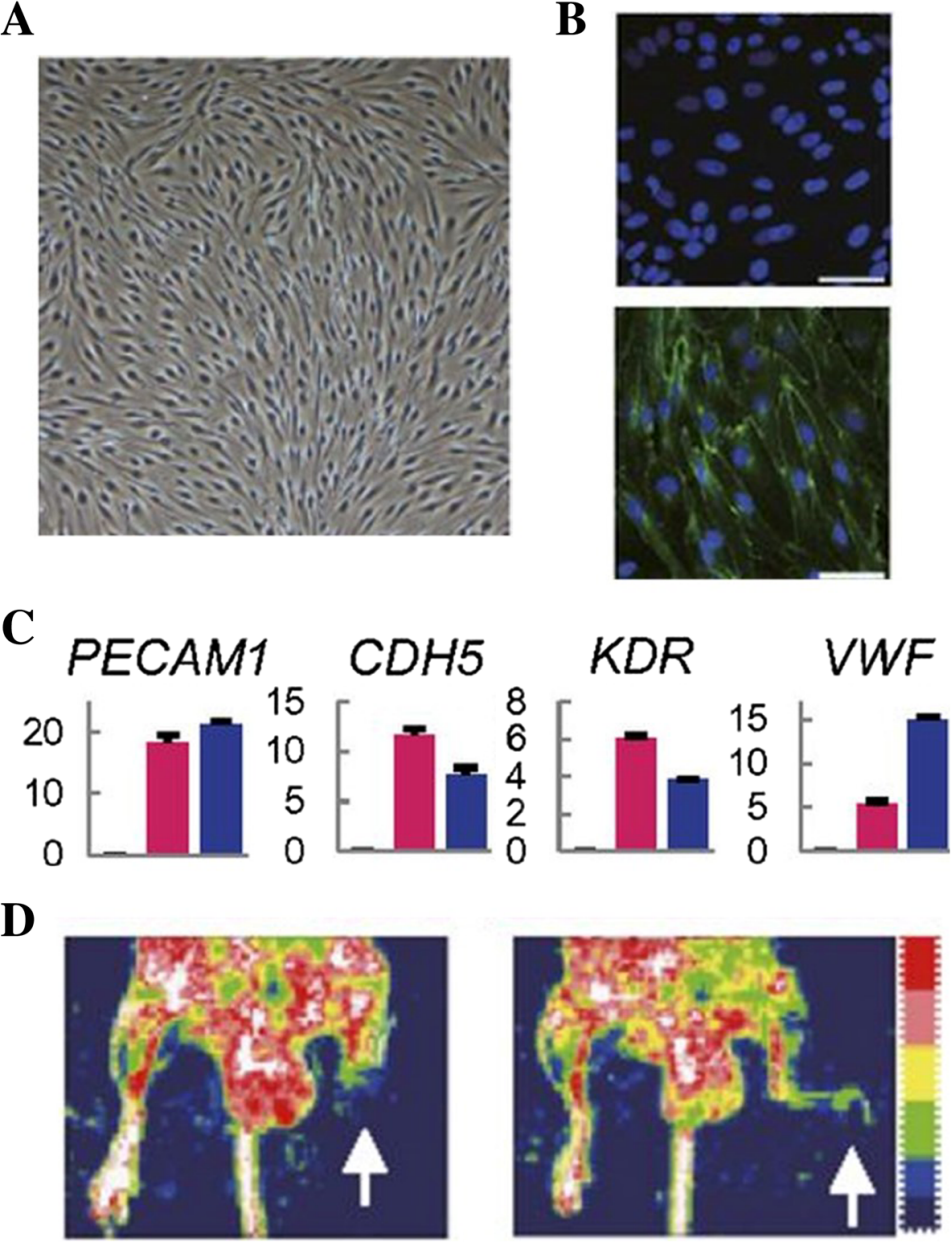
**a** ETVECs take on a typical endothelial cobblestone pattern. **b** HAFs (top) and ETVECs (bottom) stained for VE-cadherin (green). **c** qRT-PCR analysis of EC mRNA markers of fibroblasts (black), ETVECs (pink), and HUVECs (blue). **d** Hind limb ischemic mice treated with HAFs (left) and ETVECs (right) [4]

Cardiovascular disease is the leading cause of death worldwide and often results in blood vessel damage. Blood vessels are difficult to replace, and the current methods of autografts, allografts, xenografts and iPSCs all have limitations. Patients have a limited supply of blood vessels to use as autografts, allografts and xenografts can potentially cause negative immune responses, and iPSCs have the potential for tumor formation [52]. As such, a search for an external source of vasculature has risen to the forefront of research. Morita et al. (2014) converted human adult fibroblasts (HAFs) into endothelial-like cells (ETVECs) by using lentiviral transgenes to overexpress ETV2, a TF responsible for the early development of endothelial cells [4, 53, 54]. Interestingly, they found that an intermediate induction of ETV2 allowed for the best reprogramming efficiency, showing that too much ETV2 can have a negative impact on the conversion process. Overexpressing ETV2 led to the development of a typical endothelial “cobblestone” morphology roughly 41 days after induction (Fig. 5a). The reprogrammed cells were stained for VE-cadherin (Fig. 5b), which is vital for the functioning of typical endothelial cells [4, 54, 55]. The overexpression of ETV2 caused a cascade of other endothelial-specific mRNA markers to be heavily upregulated (Fig. 5c). These cells were isolated and cultured for an extended period of time, during which they maintained their commitment and functionality. The cells were then inserted into hind limb ischemic mice to view their angiogenic, vasculogenic, and overall regenerative properties. The reprogrammed fibroblasts prevented necrosis in the mice, as well as promoted revascularization (Fig. 5d) [4].

Schachterle et al. (2017) investigated the role of Sox17 in amniotic-to-endothelial cell transdifferentiation. The resulting cells had excellent engraftment properties, allowing them to integrate well with a host’s preexisting vasculature network [26]. Unfortunately, cells reprogrammed with Sox17 showed incomplete transdifferentiation and cells reprogrammed with ETV2 had low conversion efficiencies [26, 56].

#### Skeletal myocytes

**Fig. 6:**
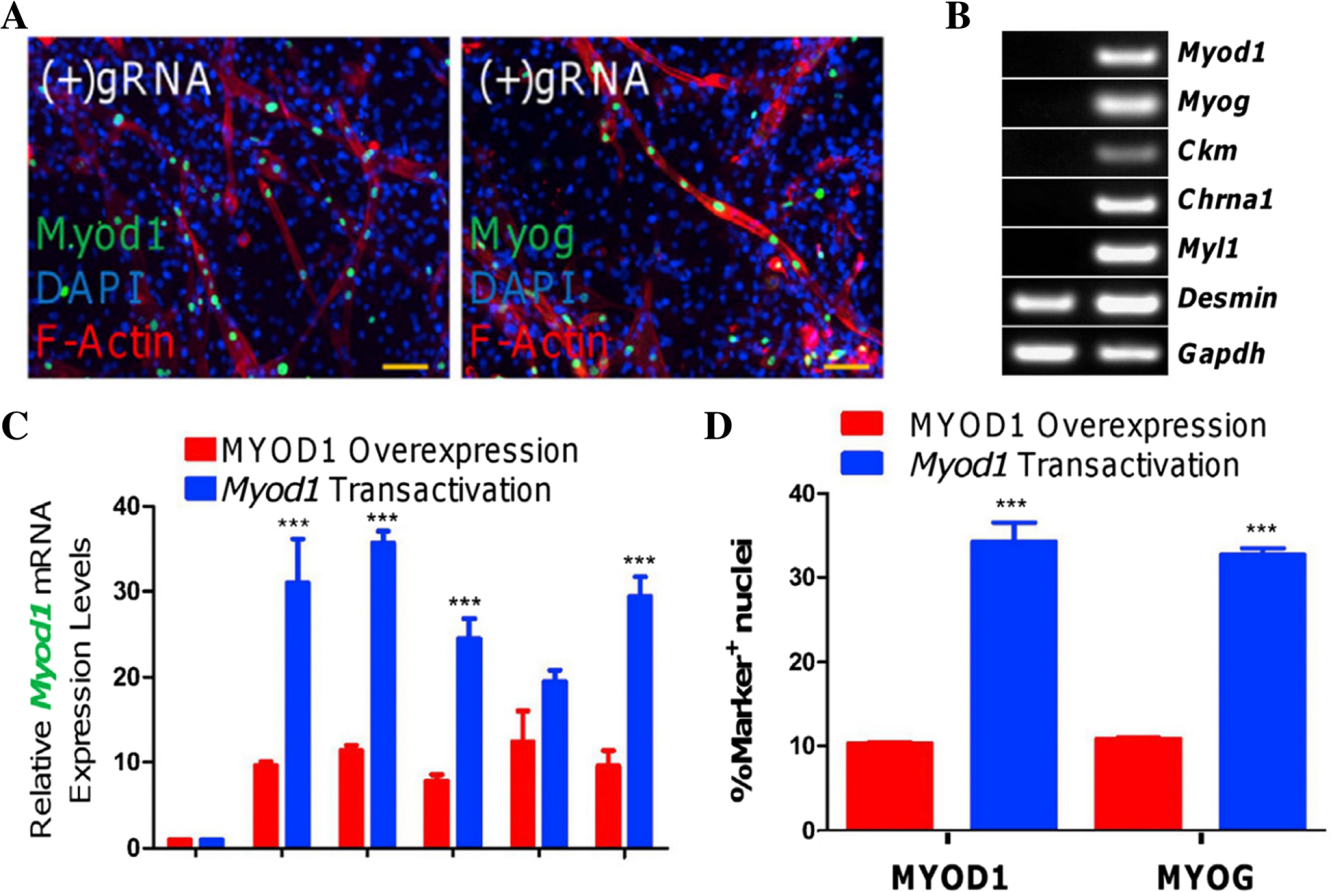
**a** Reprogrammed cells stained for nuclear Myod1 and Myog. **b** Western Blot of skeletal myocyte proteins found in untreated fibroblasts (left) and reprogrammed cells (right). **c** Myod1 levels after induction is stopped in the transgenic model (red) and dCas9 system (blue). **d** Percentage of cells that express Myod1 or Myog in the transgenic model (red) and dCas9 system (blue) [8]

Mentioned briefly above, Chakraborty et al. (2014) used dCas9 to create functional skeletal myocytes by upregulating endogenous Myod1 in fibroblasts [8]. The induced myocytes began to show myotubule formation, a hallmark of skeletal myocytes (Fig. 6a). The induction of Myod1 was correlated with the upregulation of other skeletal myocyte protein markers (Fig. 6b). In addition, the cells remained committed to their skeletal myocyte lineage after dCas9 was no longer artificially promoting the transcription of Myod1(Fig. 6c), more so than when Myod1 was transgenically overexpressed [8, 57]. The same research group converted fibroblasts into skeletal myocytes using lentiviral vectors to overexpress Myod1, and the dCas9 method produced a higher percentage of Myod1^+^ and Myog^+^ cells, implying that the dCas9 system resulted in a greater conversion efficiency (Fig. 6d) [58].

Boularaoui et al. (2018) investigated the effect of select media supplements and ECM compositions on the fibroblast to skeletal myocyte reprogramming process. Signaling pathways that are responsible for regulating myogenesis and skeletal muscle regeneration were targeted. As such, the fibroblasts were subject to TGFβ inhibition, WNT signaling activation, EGF, and IGF1, all of which promoted a significant increase in transdifferentiation efficiency and yield [58, 59]. Tissue culture plastic coated with Type I collagen, laminin, or fibronectin also resulted in an increase in transdifferentiation efficiency by promoting cell proliferation, migration, and reprogramming [60].

#### Chondrocytes

Dermal fibroblasts are a favorable cell choice when attempting to generate chondrocytes, as they have mesenchymal origins, readily proliferate, and actively produce large amounts of extracellular matrix. Dermal fibroblasts are able to undergo chondrogenic differentiation when cocultured with mature chondrocytes. Yin et al. (2010) cultured dermal fibroblasts with soluble cartilage-derived morphogenetic protein 1 (CDMP1), a protein vital in the early stages of limb chondrogenesis [61]. Over a week, cells treated with CDMP1 gradually shifted from a long spindle morphology, typical of fibroblasts, into a polygonal shape resembling chondrocytes. Several chondrocyte-specific markers were upregulated, including aggrecan, Sox9, and Type II collagen [62]. Interestingly, these cells did not maintain their phenotype when cultured in a monolayer but remained committed when subjected to micromass or pellet culture [61].

#### Pancreatic cells

Endocrine β-cells are responsible for the storage and release of insulin, making them a potential therapy for patients with Type 1 diabetes. The current supply of transplantable β-cells is far too short, making them unfeasible for use Type 1 diabetes treatments [63]. However, exocrine cells could potentially be used as a cell source for transdifferentiated β-cells. Zhou et al. (2008) generated β-like cells in situ by expressing three key transcription factors in mice pancreases. The reprogrammed cells resembled the shape, size, and ultrastructure of β-cells. PCR analysis revealed that they also expressed several genes that are essential for β-cell functions, as well as secreted insulin to regulate blood glucose levels [64].

Lemper et al. (2015) generated β-like cells by transducing human adult exocrine cells with lentiviral vectors coding for MAPK and STAT3 [65]. MAPK and STAT3 overexpression caused a large upregulation in neurogenin 3, a transcription factor that drives undifferentiated pancreatic cells towards the β-cell lineage and upregulates many other endocrine markers [66]. Furthermore, culturing the cells in a 3D matrix of Matrigel increased the efficiency of the transdifferentiation process, likely by increasing cell-cell contact. When these cells were engrafted in immunocompromised mice, they successfully produced insulin and acquired select functions of β-cells, marked by the increased expression of proteins vital to the regulation of blood glucose levels [65].

## Applications

### Tissue engineering

Margariti et al. (2012) have had success with using transdifferentiated endothelial cells as a cell source for decellularized vascular scaffolds [25]. The scaffolds were seeded with the reprogrammed cells and placed in a bioreactor with pulsatile flow to imitate physiological conditions. These cells expressed key endothelial adhesion proteins, formed vascular lumen, and resembled a typical endothelial morphology. However, these vascular grafts do not use smooth muscle cells; smooth muscle cells are vital to ensure the proper structure and function of the graft should it ever see use in in vivo applications. Smooth muscle cells are easier to acquire than endothelial cells, but if the smooth muscle cells are not from the same host as the reprogrammed endothelial cells, there is a potential for an unfavorable immune response [67]. Hong et al. (2017) generated functional endothelial cells from smooth muscle cells, and seeded a decellularized vascular graft with the original smooth muscle cells on the exterior and the reprogrammed endothelial cells on the interior [68]. When cultured in a bioreactor, the reprogrammed endothelial cells formed a complete monolayer and the surrounding layers of smooth muscle cells maintained blood pressure and vessel homeostasis, demonstrating the graft’s ability to emulate physiological vasculature [69]. These grafts show great promise for future uses in tissue engineering, due to the low risk of immune rejection and tumorigenesis.

Reprogrammed hepatocytes have been successfully used in regenerating livers in mice [48]. Ni et al. (2016) focused on improving the functionality of these cells to make them a more viable option for use in humans. They developed a method to create transdifferentiated hepatocytes that are highly effective at biosynthesizing and excreting bile acid, which are necessary for healthy liver function. Previous reprogrammed hepatocytes failed to produce bile acid. The generation of bile acid could allow for the treatment of cholestatic diseases, where the liver is unable to move bile to the small intestine on its own. Thus, this opens the door to treat more liver diseases outside of strictly liver damage [70].

### Regenerative medicine

Cells generated using transdifferentiation are generally created because the desired cell type has proliferation limitations, are found in a limited supply in the body, or are difficult to create using other methods. The most appealing cell type from transdifferentiation is neuronal cells, as they fall under all three of the aforementioned categories. However, transdifferentiated neuronal cells will likely experience some difficulty in receiving approval for clinical applications due to the lentiviruses used to create them. Typically, neurodegenerative disorders arise due to defects in neural or glial cells found in the brain and spinal cord, leading to diseases such as Parkinson’s and strokes [71]. The cause of Parkinson’s can be traced to the death or breakdown of dopamine-producing neurons in the brain. As dopamine levels in the brain fall, the brain’s activity becomes abnormal, leading to Parkinson’s disease [72]. Neural stem cells derived from Sertoli cells were found to significantly increase the function of dopaminergic neurons as well as show positive therapeutic effects when implanted into a Parkinson’s mouse model [73]. The most common type of strokes, ischemic strokes, occur when a blood vessel in the brain becomes blocked. Neural stem cells derived from embryonic fibroblasts were injected into the cortex of a stroke mouse model. The cells reduced the size of the lesion as well as promoted the recovery of fine motor and sensory functions [74].

**Fig. 7:**
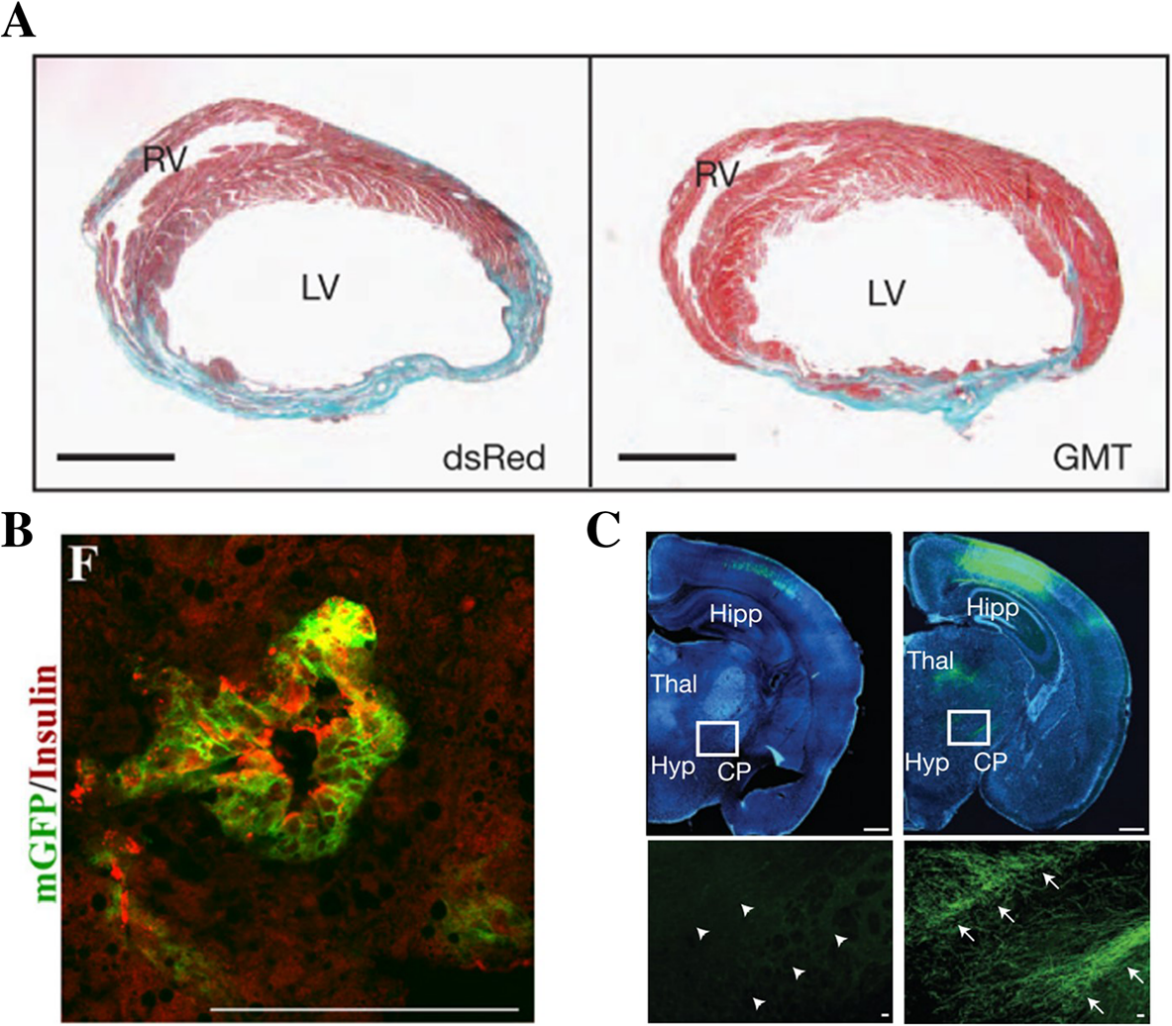
**a** Cross-sections of murine hearts depicting scar area (blue) and healthy tissue (red), in a control (left) or with transcription factors (right) [61]. **b** Insulin secretion from transdifferentiated Sox9^+^ cells [69]. **c** Axon propagation in the cerebral peduncle area in a control (left) or with Fezf2 (right) [70]

In situ treatments of specific diseases are intriguing, as it removes the need to conduct transdifferentiation outside of the body. As such, multiple research groups have been successful in localized in situ transdifferentiation to potentially treat various ailments. Cardiac fibroblasts located in the heart have been transdifferentiated into induced cardiomyocytes capable of improving cardiac function after a myocardial infarction in murine models (Fig. 7a) [75]. Adenoviruses were used to transdifferentiate Sox9^+^ cells, commonly found in the small bile ducts around the liver, into insulin-producing cells that were able to combat diabetes in the long-term in mice (Fig. 7b) [76]. Postnatal callosal projection neurons present in the corpus callosum were transdifferentiated in situ into corticofugal neurons via the overexpression of transcription factor Fezf2 (Fig. 7c) [77].

## Challenges with clinical translation and potential solutions

There are several major difficulties associated with using transdifferentiated cells in clinical applications. The most glaring issue is the use of lentiviruses to infect cells, due to the small possibility of unintended insertional mutagenesis [78]. These mutations, while unlikely, could cause drastic, unforeseen consequences in the host, such as the emergence of cancer [79]. Understandably, many government agencies take precaution due to this risk. Non-integrating viruses and other methods that do not integrate DNA into the host genome do not pose these threats, but have much lower reprogramming efficiencies. Therefore, there is a need to efficiently transdifferentiate cells while avoiding the possibility of mutagenesis. The advent of dCas9 allows for a drastic reduction of the chance of mutagenesis through its ability to multiplex. When lentiviral vectors are used to overexpress multiple exogenous transcription factors, more than one vector may be used due to the cargo capacity limitations of lentiviruses. However, transdifferentiation methods utilizing dCas9 only need to use one vector to efficiently express the dCas9. Once the cells express dCas9, several gRNAs targeting various genes can be added through non-integrating methods, allowing the dCas9 to regulate the expression of several genes despite the cells receiving a single lentivirus infection [80]. Thus, reducing the number of DNA-integrating viruses needed to transdifferentiate cells lowers the chance for insertional mutagenesis. Another alternative that would completely remove the potential for mutagenesis would be through the delivery of dCas9/gRNA Ribonucleoprotein complexes (dCas9 RNPs). dCas9 RNPs consist of dCas9 preloaded with a gRNA, which are then directly delivered to cells using electroporation or transfection techniques, eliminating the need for DNA integration into the genome. However, dCas9 RNPs come with a major drawback; they are cleared rapidly from the cell through protein degradation pathways [81]. Therefore, the dCas9 RNPs would need to be reintroduced into the source cells at regular intervals in order to effectively transdifferentiate the cells.

Another concern with transdifferentiated cells is their ability to completely mimic their desired cell phenotype, as it is likely that the transdifferentiated cells will not be identical to their native counterparts. Thus, more complete reprogramming processes are needed, in order to generate transdifferentiated cells that more closely resemble the desired cell phenotype. Through thorough testing and experimentation, the major characteristics of the reprogrammed cells can be analyzed and compared to native cells. Although in vitro assays will analyze some of the reprogrammed cells’ properties, well-designed in vivo assays are necessary to fully characterize them in a physiological setting. Current in vivo studies are superficial and typically fail to detail more than a handful of reprogrammed cell capabilities; as such, more extensive testing in animal models is necessary before transdifferentiated cells see any translation to clinical applications.

Lastly, reprogramming efficiency is another problem associated with the transdifferentiation process. A low conversion efficiency generally leads to a lengthy period of time before there are enough reprogrammed cells for any clinical application, hindering the use of transdifferentiated cells in humans, as clinical situations are often time-sensitive. Consequently, improving the efficiency and cell yield of the transdifferentiation process is vital in order to make transdifferentiation more favorable for clinical applications. This can be done with a myriad of methods, which include optimizing biochemical [82], biophysical [83], and biomechanical [84] cues the cells experience during the reprogramming process, targeting additional transcription factors, and transitioning from exogenous overexpression to endogenous upregulation via dCas9.

## Summary

Transdifferentiation is a powerful tool for generating functional cell phenotypes without the need for iPSCs or embryonic stem cells. Over the past several years, several techniques for cellular reprogramming have been developed and various targeted cell phenotypes have been generated, with encouraging results. Although current transdifferentiation methods are somewhat limited due to efficiency problems, there is ongoing research that aims to improve efficiency and there has been preliminary success with the emergence of dCas9 as an alternative to transgene overexpression methods. Regardless of efficiency limitations, a wide array of cells has been successfully generated and their ability to mimic physiological cells shows great promise, especially with the advent of transdifferentiating cells in situ. These cells still have a long way to go to achieve fully functional states and see use in tissue engineering, as rigorous clinical testing needs to be conducted. Nevertheless, considering how infantile the fields of reprogramming and transdifferentiation are, it would not be surprising to see transdifferentiated cells have a place in personalized regenerative medicine and tissue engineering in the future.
